# The Larval Zebrafish Vestibular System Is a Promising Model to Understand the Role of Myelin in Neural Circuits

**DOI:** 10.3389/fnins.2022.904765

**Published:** 2022-05-06

**Authors:** Franziska Auer, David Schoppik

**Affiliations:** Departments of Otolaryngology, Neuroscience & Physiology, Neuroscience Institute, New York University Grossman School of Medicine, New York, NY, United States

**Keywords:** myelin, zebrafish, vestibular, neural circuit, development

## Abstract

Myelin is classically known for its role in facilitating nerve conduction. However, recent work casts myelin as a key player in both proper neuronal circuit development and function. With this expanding role comes a demand for new approaches to characterize and perturb myelin in the context of tractable neural circuits as they mature. Here we argue that the simplicity, strong conservation, and clinical relevance of the vestibular system offer a way forward. Further, the tractability of the larval zebrafish affords a uniquely powerful means to test open hypotheses of myelin's role in normal development and disordered vestibular circuits. We end by identifying key open questions in myelin neurobiology that the zebrafish vestibular system is particularly well-suited to address.

## 1. Introduction

Myelin's primary claim to fame is as the structure that enables fast nerve conduction, but as methods and techniques have improved, a variety of findings have expanded myelin's role. We now know that oligodendrocytes and Schwann cells provide metabolic support (Fünfschilling et al., 2012; Lee et al., 2012). Further, myelin plays a crucial role for neural circuit function and plasticity, particularly during development (Makinodan et al., 2012; McKenzie et al., 2014; Xiao et al., 2016; Steadman et al., 2020). New myelin is crucial for learning new tasks (McKenzie et al., 2014; Xiao et al., 2016) as well as for memory consolidation (Pan et al., 2020; Steadman et al., 2020). Social isolation leads to myelin deficits and impaired social behavior (Liu et al., 2012; Makinodan et al., 2012) that can be rescued by promoting myelin (Liu et al., 2016). Illuminating the mechanistic underpinnings of these new roles will require expanding the current toolkit to permit functional assessment in combination with high resolution imaging and control of myelination (Suminaite et al., 2019).

The vestibular circuits of zebrafish offer a number of advantages as a model system. First and foremost both the mechanisms regulating myelination (Czopka, 2015; Ackerman and Monk, 2016) and vestibular circuits are conserved between zebrafish and mammals (Straka and Baker, 2013). Secondly, there is a large tool set to targe myelin (Chung et al., 2013; Auer et al., 2018; Neely et al., 2022) and to control neurons in vestibular circuits (Ehrlich and Schoppik, 2017, 2019; Schoppik et al., 2017). Due to their transparency, zebrafish larvae can be imaged longitudinally without the need for surgical interventions. Furthermore, vestibular circuits can be tested behaviorally as well as electrophysiologically to assess changes in conduction and neuronal function (Bagnall and Schoppik, 2018; Ehrlich and Schoppik, 2018). Vestibular function improves over time (Ehrlich and Schoppik, 2017, 2019), allowing for correlation of behavior with myelination during development (Langworthy, 1928, 1932; Keene and Hewer, 1931) and in the context of disease (Doty et al., 2018).

Here, we begin by highlighting recent advances in myelin biology in the context of neural circuits relevant for vestibular scientists. We then discuss the organization of vestibular circuits with particular emphasis on the establishment and organization of myelinated tracts that subserve balance behaviors of particular interest for zebrafish scientists. Next, we review the consequences of demyelinating diseases on balance behavior. We highlight recent advances in zebrafish vestibular circuits, behavior and myelin development of interest for myelin researchers interested in vestibular circuits. We conclude by identifying open questions in myelin biology addressable using the larval zebrafish vestibular system as a model.

## 2. Myelination and Circuit Function

Myelin is a fatty and electrically insulating substance that is wrapped around axons. Myelin sheaths are interspersed with unmyelinated gaps, the nodes of Ranvier, which are enriched in voltage-gated sodium channels, among other molecules. This arrangement restricts current flow to the nodes of Ranvier enabling the fast saltatory nerve conduction and thereby increasing conduction velocity. Myelin sheath length, myelin thickness (Seidl, 2014) and node length (Arancibia-Cárcamo et al., 2017) influence conduction velocity. Besides being faster, saltatory nerve conduction is also more efficient; energy consumption in gray matter is roughly three times higher than in white matter (Sokoloff et al., 1977), reflecting both the efficiency of saltatory nerve conduction and the reduced synaptic density of white matter (Harris and Attwell, 2012). Myelinating cells also actively provide metabolic support to the ensheathed axons (Fünfschilling et al., 2012; Lee et al., 2012; Beirowski et al., 2014). Lactate is transported via monocarboxylate transporters from the oligodendrocyte to the ensheathed axon thereby supporting axonal function. Genetic disruption of the lactate transportation pathway may lead to axonal degeneration (Fünfschilling et al., 2012; Lee et al., 2012) and affect auditory processing (Moore et al., 2020).

**Figure 1:**
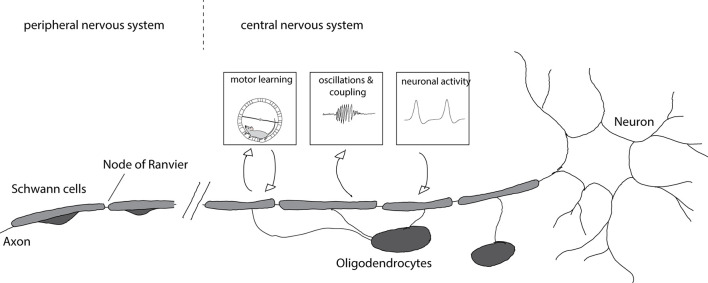
Myelination of axons and consequences for circuit function. Axons in the central nervous system are myelinated by oligodendrocytes whereas axons in the peripheral nervous system are myelinated by Schwann cells. Myelination is enhanced after motor learning and the newly formed myelin is necessary for learning. Furthermore, newly formed myelin is implicated in increased coupling between cortical spindle oscillations and hippocampal sharp wave ripples that affect memory formation. Enhanced neuronal activity after chemogenetic or optogenetic stimulation results in increased myelin formation and thicker myelin sheaths.

Myelinating cells in the peripheral nervous system are different from those in the central nervous system. Oligodendrocytes are the myelinating cells of the central nervous system (CNS) whereas Schwann cells myelinate axons in the peripheral nervous system (PNS). Axons with central and peripheral projections are myelinated by oligodendrocytes on the central part and Schwann cells on the peripheral part (Figure 1).

During development oligodendrocyte precursor cells (OPC) differentiate into oligodendrocytes, forming many myelin sheaths on several different axons. In contrast, Schwann cells envelop many axons in their immature state and, after radial sorting, form a myelin sheath around a single axon. There are also non-myelinating Schwann cells that envelope many smaller axons (Jessen and Mirsky, 2005). The differences in the mechanisms of myelination in the PNS and CNS have been reviewed elsewhere (Nave and Werner, 2014).

The impact of myelin on axons is not unilateral (Figure 1). Recent work provided evidence that axonal activity can also regulate myelination: OPC proliferation and differentiation increase after optogenetic activation of neurons in the premotor cortex leading to increased myelination, thicker myelin around stimulated axons, and alterations in motor function (Gibson et al., 2014). Similarly, chemogenetic activation of neurons results in enhanced myelination and myelin thickness, preferentially of stimulated axons (Mitew et al., 2018). Such activity-dependent myelination is thought to be mediated by synaptic activity. OPCs form synaptic contacts with axons (Bergles et al., 2000) and axonal activity regulates their proliferation (Barres and Raff, 1993). Blocking vesicle release results in decreased numbers of myelin sheaths per oligodendrocyte (Mensch et al., 2015), though this effect may be restricted to particular types of neurons (Koudelka et al., 2016).

Intriguingly, a number of recent studies have linked myelination to key aspects of learning and memory formation. Learning a new motor task—like running on a complex wheel—causes differentiation of oligodendrocyte precursor cells and the formation of new myelin in mice; when myelin formation was blocked, learning was impaired (McKenzie et al., 2014; Xiao et al., 2016). Complementarily, motor learning promoted recovery of myelin in a mouse model of cuprizone induced demyelination (Bacmeister et al., 2020). Spatial learning and contextual fear learning is also impaired when oligodendrogenesis is blocked (Pan et al., 2020; Steadman et al., 2020). Increased coupling of cortical spindle oscillations and hippocampal sharp wave ripples is important for memory consolidation (Peyrache et al., 2009; Xia et al., 2017). Preventing oligodendrogenesis interferes with this coupling, implicating the formation of new myelin (Steadman et al., 2020). Conversely, age related memory decline in mice could be rescued by promoting myelination (Wang et al., 2020), further emphasizing important roles for oligodendrogenesis and the formation of new myelin for learning and memory.

There are several ways in which newly added myelin can influence neural circuits. New myelin could cause specific changes to synchronicity or the timing of transmission making neural circuits more efficient. It has been shown that some axons display intermittent myelination (Tomassy et al., 2014). These large unmyelinated gaps could serve as potential targets/locations where adaptive myelination could sculpt the circuit. Small changes in myelination resulting in small changes in conduction velocity can significantly alter brain oscillations (Pajevic et al., 2014). Consistently, inhibitory parvalbumin-positive interneurons, which are important for oscillations and modulating oscillations have been found to be frequently myelinated (Stedehouder et al., 2017) further indicating that myelination might be important for brain oscillations and synchronicity.

## 3. Functional Organization of Vestibular Reflex Circuits and Their Myelinated Tracts

The vestibular system serves a vital purpose: to stabilize posture and gaze in response to destabilizing forces. To do so, it produces corrective behaviors in response to body/head motion (Goldberg et al., 2012). Functional simplicity and well-characterized anatomy have made vestibular reflexes exceptional models for understanding basic principles of neural circuit function. Vestibular reflexes fall into two primary categories: vestibulo-ocular reflexes stabilize gaze, and vestibulospinal/vestibulocolic reflexes stabilize posture. The critical neural circuit underlying each reflex consists of three classes of neuron: (1) vestibular ganglion neurons that relay transduced forces to (2) central brainstem neurons that in turn relay commands to (3) motor neurons that produce compensatory muscle contractions. Connecting these three classes of neuron are axon tracts. The majority of axons that comprise vertebrate vestibular tracts are myelinated (Scherer and Easter, 1984; Fermin and Igarashi, 1987; Fraher, 1989a; Berardinelli et al., 2000). Intriguingly, tracts transmitting vestibular information are some of the first to be myelinated in mammals during development (Langworthy, 1932) and the occurrence of myelin maybe correlated with the development of specific abilities (Langworthy, 1928, 1932; Keene and Hewer, 1931).

**Figure 2:**
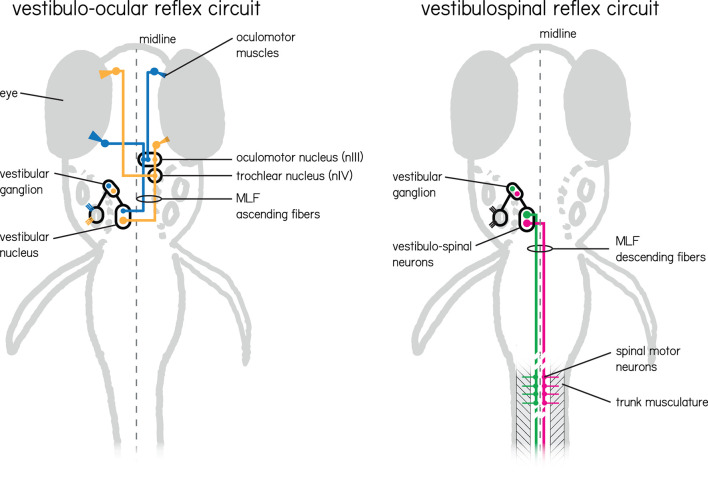
Two vestibular circuits in the larval zebrafish responsible for stabilization of gaze and posture. (Left) The vestibulo-ocular reflex circuit is responsible for stabilizing gaze. Illustrated in color are the primary populations responsible for the vertical and torsional directions of rotation. Nose-up/eyes-down are illustrated in yellow, and nose-down/eyes-up are illustrated in blue. Both channels sense instability with hair cells in the inner ear tuned to a particular direction of linear acceleration. These hair cells relay information to VIIIth nerve vestibular afferent neurons located in the vestibular ganglion, and then in turn to neurons in the vestibular nuclei. Individual vestibular nucleus neurons sent ascending projections along the medial longitudinal fasciculus (MLF) to either cranial nucleus III alone (blue) or cranial nucleus III and IV (yellow) where motor neuron somata send axons to their respective eye muscles. (Right) The vestibulospinal circuit has similar peripheral input, but instead of ascending projections from vestibular nucleus neurons leverages descending projections that also run along the medial longitudinal fasciculus. These neurons (green and pink) comprise the lateral and medial vestibulospinal tracts and project to spinal interneurons and motor neurons which in turn control trunk musculature.

Here we detail the organization of vestibular circuits (Figure 2), with particular focus on myelination.

Instability is transduced into neural activity by hair cells located in two types of sense organs located in the inner ear. First, the otolithic organs—the utricle and saccule—sense linear accelerations (e.g., head tilts or body translations) in the horizontal and vertical axes, respectively. Second, three semicircular canals detect angular acceleration (e.g., head/body rotations) in the pitch, yaw and roll axes. Neurons in the vestibular ganglion are bipolar, with both a peripheral and central projection. The peripheral projection extends from the somata to synapse on the hair cells on each of the vestibular end organs. The centrally-projecting axons comprise branches of the VIIIth cranial nerve, entering the brainstem at the level of the lateral vestibular nucleus and terminating in the vestibular nuclei and the cerebellum (Büttnrt-Ennever, 1992). Finally central vestibular efferent neurons send projections from the brainstem to the periphery as part of the VIIIth nerve, where they are thought to impact sensation (Mathews et al., 2017; Raghu et al., 2019).

In mammals, most axons in the VIIIth nerve are myelinated, and the somata of vestibular ganglion neurons are myelinated as well (Fermin and Igarashi, 1987). Like many cranial nerves, there is a bimodal distribution of fiber diameter in the VIIIth nerve that emerges during development, suggesting that neurons are myelinated at different times (Bronson et al., 1978; Kerns, 1980; Hahn et al., 1987; Fraher, 1989a,b; Bardosi et al., 1990; Berardinelli et al., 2000). Larger axons are myelinated first followed by thinner ones during later stages of development. While peripheral branches of the vestibular nerve innervate different sensory structures, their fiber diameters, numbers, and myelination are comparable (Landolt et al., 1973). In contrast, vestibular efferents are thin with predominantly unmyelinated axons (Raghu et al., 2019). Finally, the vestibular nerve also contains some unmyelinated fibers in close contact with blood vessels thought to have vasomotor function (Rasmussen, 1940). Post-mortem studies revealed that the vestibular nerve in humans is already heavily myelinated at birth (Bergström, 1973). There is a slight loss of myelinated fibers during aging, which is most apparent in the late 70s (Rasmussen, 1940; Bergström, 1973).

Myelin development in the VIIIth nerve proceeds along a peripheral to central gradient, with implications for functional development. In mice, at the 15th and 16th gestational day Schwann cells start to enclose vestibular nerve axons. By postnatal day 4 the peripheral part of the vestibular nerve is myelinated whereas the central part shows less signs of myelination. By postnatal day 10 the amount of myelination of the peripheral part has reached adult values (Anniko, 1985). Vestibular ganglion somata are also myelinated, however this occurs later, around 7–21 days postnatally (Dechesne et al., 1987). During this post-natal period, responses of vestibular neurons mature (Desmadryl, 1991). The gradient in myelination in vestibular ganglion neurons is reminiscent of findings in the cochlear branch of the VIIIth nerve in humans. There, the central projection is myelinated 2–3 weeks after the peripheral projection, with concomitant functional maturation (Moore and Linthicum, 2001).

Central axons of vestibular ganglion neurons project as part of the VIIIth nerve to the brainstem and cerebellum. Vestibular brainstem nuclei are named for their relative locations in the hindbrain: medial, lateral, superior, and inferior; anamniotes and birds have a fifth, called the tangential nucleus. Projections from these vestibular nuclei that subserve reflexive behavior can be categorized with respect to their target motor pools. The vestibulo-ocular reflex relies on three cranial motor nuclei: the oculomotor (nIII), the trochlear (nIV), and the abducens (nVI) that collectively stabilize gaze in the pitch, roll, and yaw axes (Dickman, 2018). The vestibulospinal/vestibulocolic reflexes maintain muscle tone and produce corrective movements through alpha/gamma motor neurons of the spinal cord (Wilson, 1995; Goldberg and Cullen, 2011).

Brainstem vestibular axons project along three major tracts: the medial longitudinal fasciculus (MLF), the lateral vestibulospinal tract (LVST), and medial vestibulospinal tract (MVST). The MLF contains ascending and descending fibers. The ascending fibers connect the vestibular nuclei to cranial motor nuclei (Watson, 2012). Ascending neurons with ipsilateral axons tend to release inhibitory neurotransmitters, while those that cross the midline are predominantly excitatory. The MVST runs within the MLF and innervates motor neurons of the neck muscles of the ipsilateral and contralateral cervical spinal cord (Kasumacic et al., 2010; Rea, 2015; Lambert et al., 2016). The lateral vestibulospinal tract (LVST) innervates alpha and gamma motor neurons at all levels of the ipsilateral spinal cord and interneurons in laminae VII–IX (Kasumacic et al., 2015; Dickman, 2018). Notably, forelimb motor neurons receive only LVST input (Lambert et al., 2016). Neurons in the LVST are excitatory whereas neurons from the MVST can be either excitatory or inhibitory (Dickman, 2018).

In rats, the MLF has only a small amount of myelin at birth but this increases rapidly and at 21 days the myelination is comparable to adult levels (Hamano et al., 1998). In humans the MLF is one of the earliest central tracts where myelin staining appears; it is visible around the 14th week of gestation (Keene and Hewer, 1931), reaching adult levels at 34 weeks of gestation. This early myelination is proposed to be important for the earliest vestibular reflexes of the head, neck and upper extremities (Gilles, 1976; Tanaka et al., 1995).

The final target of the vestibulo-ocular reflex, the extraocular muscles, are innervated by three cranial nerves. The oculomotor nerve (cranial nerve III) innervates the medial, superior and inferior rectus and the inferior oblique muscle. The trochlear nerve (cranial nerve IV) innervates the superior oblique muscle, and the abducens nerve (cranial nerve VI) the lateral rectus muscle (Purves, 2004). The majority (80–90%, depending on the species) of axons in these nerves are myelinated (Scherer and Easter, 1984; Fraher, 1989a; Berardinelli et al., 2000). In sheep, the remaining unmyelinated fibers are enveloped by Schwann cells, and may play a sensory role (Berardinelli et al., 2000). Analysis of the timeline of myelination of the VIth cranial nerve in rats showed that there is a rapid increase in myelinated fibers in the first postnatal week. In the following week myelination seems to halt, whereas in the third postnatal week the number of myelinated fibers increase again as thin axons become myelinated (Hahn et al., 1987). A similar progression has been described for the trochlear nerve in rats (Kerns, 1980).

Extraocular muscles contain both fast- and slow-twitch fibers innervated by similarly specialized motor neuron axons. Axon diameter is bimodally-distributed within the ocular motor nerves (Bronson et al., 1978; Fraher, 1989a,b; Bardosi et al., 1990; Berardinelli et al., 2000). Small diameter axons innervate the slow muscle fibers, whereas the large diameter axons innervate the fast muscle fibers of the extra ocular muscles (Dietert, 1965; Browne, 1976). Myelin thickness generally correlates with axon diameter. However, in human abducens nerve, [narrow/wide] axons are found with both thick and thin myelin. There, as in the vestibular periphery, the difference in myelin thickness is thought to reflect differential onset of myelination (Bardosi et al., 1990).

The cerebellum plays a key role in modulating vestibular reflexes (Lance, 1981). Inputs to the cerebellum arise directly from the vestibular afferents (Balmer and Trussell, 2019), and Purkinje axons pass through the juxtarestiform body and connect the vestibular nuclei with the cerebellum (Dickman, 2018). In mice fibers of the juxtarestiform body show faint myelin staining in the newborn which increases gradually and are heavily myelinated by end of the second postnatal week (Bernstein, 1957). The vestibular lobes, flocculonodular lobe and parafloccular lobe, of the cerebellum show myelin staining toward the end of the 2nd postnatal week while the other lobes of the cerebellum already show myelin staining at the beginning of the second postnatal week (Bernstein, 1957). In humans, myelination starts around 16 weeks gestation and the cerebellar tract is heavily myelinated at birth (Keene and Hewer, 1931).

Vestibular reflex circuits are a powerful model for understanding neural development and function due to their well-defined anatomy. Further, we have a broad understanding of the timeline of myelination, particularly as it relates to the onset of function. While particular timelines may vary between different species, they are broadly correlated with reflex capacity at birth (Langworthy, 1932). Consequentially, vestibular reflex circuits are well suited to assess the functional consequences of myelination during development and demyelination during disease.

## 4. Demyelinating Diseases and Their Effects on Balance

Multiple Sclerosis (MS) is a demyelinating autoimmune disease with a broad range of symptoms including vision problems, weakness in arms or legs or imbalance (Matsuda et al., 2011). MS patients commonly suffer dizziness and vertigo, negatively impacting quality of life (Marrie et al., 2013) and increasing the risk of falls and injuries (Peterson et al., 2008; Matsuda et al., 2011). Balance performance correlates with the damage to white matter tracts (Prosperini et al., 2013) as well as with central lesion size (Doty et al., 2018). Impaired spinal axon conduction is thought to underlie balance impairment (Cameron et al., 2008). Physical therapy focused on improving vestibular reflexes allows better management of dizziness and vertigo in MS patients (García-Muñoz et al., 2020), suggesting a role for activity dependent remyelination (Bacmeister et al., 2020). MS lesions present as white matter hyperintensities in MRI. Functional assays of vestibular reflex capacity can serve as a powerful diagnostic tool: some MS patients with balance problems have alterations in vestibular evoked myogenic potentials even in the absence of detectable white matter hyperintensities (Stadio et al., 2019).

MS patients present with characteristic impairments to vestibulo-ocular reflexes. Internuclear ophthalmoparesis is the most common disorder in MS affecting eye movements. It has two hallmarks: a slowing of the adducting eye (adduction lag) during horizontal eye movements and an impaired vestibulo-ocular reflex, primarily the vertical vestibulo-ocular reflex. Demyelination of the abducens internuclear neurons causes horizontal adduction lag, whereas demyelination of the vestibular nuclei projections causes impairments of vertical vestibulo-ocular reflex (Leigh and Zee, 2015; Serra et al., 2018). Finally, many patients with MS develop a pendular nystagmus, an oscillating eye movement that often causes blurred vision (Leigh and Zee, 2015).

In addition to MS, other demyelinating disorders can also present with characteristic impairment to vestibular reflexes. For example, Pelizeaus Merzbacher disease is a rare X-linked developmental disorder associated with abnormal myelination of the brain and spinal cord. Children with Pelizaeus Merzbacher disease show abnormal eye movements, often developing a pendular nystagmus, with varying amplitudes and frequencies among patients. Smooth pursuit and the optokinetic nystagmus are also often defective in these patients. It is thought that these abnormal eye movements are caused by defects of cerebellar processing (Huygen et al., 1992; Leigh and Zee, 2015).

## 5. Zebrafish as a Model Organism to Study Myelination in the Vestibular System

Among vertebrates one model is especially well-suited to study myelin in the context of neural circuits: the larval zebrafish. Zebrafish develop externally, and are transparent as larvae. Zebrafish are genetically accessible, allowing molecular control of defined populations of cells. Consequentially, *in vivo* longitudinal imaging studies are routine. Crucially, the molecular and cellular mechanisms involved in myelination are conserved between zebrafish and mammals (Preston and Macklin, 2014; Ackerman and Monk, 2016). Findings in zebrafish and mice routinely complement one another; for example, neuronal activity is an important driver for myelination in both the zebrafish and mouse central nervous system (Gibson et al., 2014; Hines et al., 2015; Mensch et al., 2015). Zebrafish can therefore illuminate the role of myelin in vertebrate neural circuits.

The genetic tractability of the zebrafish preparation facilitates a focus on myelin in the vestibular system. A considerable library of validated tools now exist to label and track oligodendrocytes, Schwann cells or oligodendrocyte precursor cells (Almeida et al., 2011; Czopka et al., 2013; Hines et al., 2015; Mensch et al., 2015; Auer et al., 2018; Marisca et al., 2020). Further, there are many tools to target oligodendrocytes with both coarse (Chung et al., 2013; Neely et al., 2022) and precise (Auer et al., 2018) optical manipulations. Myelination can be accelerated or delayed by using pharmacological approaches (Early et al., 2018), and candidate genes can be targeted for loss-of-function experiments (Hruscha and Schmid, 2014; Irion et al., 2014).

In addition, reagents exist to target populations of cells responsible for vestibular reflexes (Bianco et al., 2012; Schoppik et al., 2017; Ehrlich and Schoppik, 2019; Liu et al., 2020; Hamling et al., 2021; Wu et al., 2021) Consequentially, considerable strides have been made in studying the function of neurons responsible for the vestibulo-ocular reflex (Bianco et al., 2012; Schoppik et al., 2017), and vestibulo-spinal postural reflexes (Bagnall and McLean, 2014; Ehrlich and Schoppik, 2017, 2019; Liu et al., 2020, 2021; Hamling et al., 2021; Wu et al., 2021). Here, just as in many other animal models of vestibular function, the ability to parametrically present vestibular stimuli (i.e., tilts/translations) permits rigorous quantitative assessment of neuronal function. Further, whole-brain imaging allows for simultaneous measurements of neural activity during vestibular stimulation (Favre-Bulle et al., 2018; Migault et al., 2018). Taken together, the zebrafish vestibular circuits allow an exceptional view into the role of myelination in vestibular circuit function and attendant behaviors.

**Figure 3:**
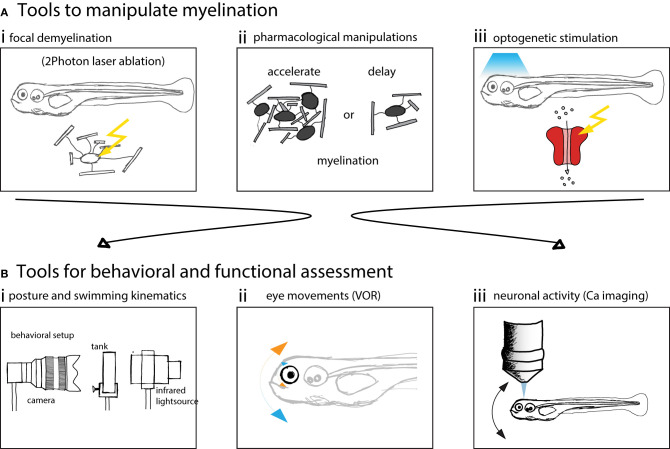
Zebrafish larvae are well suited to assess behavior and neuronal circuit function after myelin manipulations. **(A)** Schematics of possible manipulations that can be performed using zebrafish as a model system. (i) 2 Photon laser ablation can be used to selectively demyelinate and create focal myelin lesions. (ii) The development of myelination can be accelerated or delayed using pharmacological treatments. (iii) Optogenetic methods can be used to probe circuit function. **(B)** Schematics of methods used to assess vestibular behavior and circuit function. (i) Posture and swimming kinematics can be analyzed. (ii) Vestibulo-ocular reflex can be tested and compensatory eye movements analyzed. (iii) Calcium imaging can be used to measure neural activity to assess circuit function.

Here we schematized different manipulations to myelin and methods to perform behavioral or functional assessment (Figure 3).

## 6. Open Questions and Conclusions

Recent work supports the idea that newly formed myelin is important for proper neuronal circuit function and learning. Activation of axons leads to enhanced myelination or thicker myelin (Gibson et al., 2014; Mensch et al., 2015; Koudelka et al., 2016; Mitew et al., 2018) and the formation of new myelin seems crucial for new memory formation and consolidation (McKenzie et al., 2014; Xiao et al., 2016; Bacmeister et al., 2020; Pan et al., 2020; Steadman et al., 2020). Complementary, during development myelin formation allows neuronal circuits to mature. The mechanisms by which such newly formed myelin affects circuits in either development or learning are not yet understood. Here we propose that the vestibular system of the larval zebrafish will facilitate progress on three fundamental questions.

First: What role does myelin play during normal development of behavior? In both mice (McKenzie et al., 2014; Xiao et al., 2016; Bacmeister et al., 2020; Pan et al., 2020; Steadman et al., 2020) and humans (Bengtsson et al., 2005; Scholz et al., 2009; Hu et al., 2010) formation of new myelin is necessary for acquiring new skills. We hypothesize that similar mechanisms are at play during development, when many new skills are learned and improved. To date, correlations between behavioral improvements and development have primarily been inferred from post-mortem studies (Langworthy, 1928, 1932). Progress will require a system that permits longitudinal measurements of myelin progression *in vivo* such as the larval zebrafish (Almeida et al., 2011; Czopka et al., 2013; Hines et al., 2015; Mensch et al., 2015; Auer et al., 2018; Marisca et al., 2020). Similarly, both gaze-stabilizing and posture-stabilizing vestibular behaviors develop over time in the larval zebrafish (Bianco et al., 2012; Ehrlich and Schoppik, 2017, 2019; Schoppik et al., 2017; Hamling et al., 2021). Thus, the larval zebrafish is poised to permit direct longitudinal examination of the relationship between myelination and functional improvements. Crucially, libraries of reagents exist that can slow down or accelerate myelination in zebrafish (Early et al., 2018). We therefore propose that measuring and manipulating myelination in vestibular circuits of larval zebrafish while measuring postural development will allow profound insights into myelin's role during maturation.

Next: What role does metabolic support play for neural circuit function during development? Beyond its classical roles in enhancing conduction velocity and saving energy (Sokoloff et al., 1977), myelin benefits ensheathed neurons by actively providing metabolic support (Fünfschilling et al., 2012; Lee et al., 2012; Beirowski et al., 2014). Loss of this metabolic support affects auditory processing in mice (Moore et al., 2020). We hypothesize that such metabolic support will prove crucial for normal functional development of neuronal circuits. As described above, the zebrafish vestibular system is comprised of an especially accessible set of simple neuronal circuits. Crucially circuit function can be assessed following specific blockade of metabolic support, allowing powerful loss-of-function experiments. Furthermore, the sensitivity of central vestibular neurons is straightforward to assess with either optical imaging (Favre-Bulle et al., 2018; Migault et al., 2018) or electrophysiology (Liu et al., 2020; Hamling et al., 2021). These approaches will leverage the accessibility of the larval zebrafish vestibular system to understand how loss of glial metabolic support affects the integrity of developing neuronal circuits.

Finally: How do demyelination and remyelination affect vestibular circuit function? Demyelinating disorders are associated with characteristic deficits in vestibular reflexes (Leigh and Zee, 2015; Serra et al., 2018). However, demyelination is associated with inflammation and neurodegeneration making it hard to disentangle the particular consequences of demyelination from inflammatory effects. Understanding the specific behavioral consequences of demyelination is key to improve diagnosis and target therapies. Clinically, remyelination of MS lesions is often incomplete resulting in thinner and shorter myelin sheaths. The amount of remyelination also greatly varies between different patients and does not correlate with the disease course (i.e., relapsing remitting or progressive) (Patrikios et al., 2006). We therefore need a means to assess how demyelination and successful remyelination might affect circuit function. Focal demyelination is possible in zebrafish (Auer et al., 2018), and zebrafish can regenerate normal thickness myelin (Karttunen et al., 2017). If such assays were performed in vestibular circuits, it would be straightforward to assess both behavior and conduction velocity following myelin injury and recovery. We anticipate that such experiments would allow powerful insight into the mechanisms by which myelin loss and restoration can impact circuit function.

In summary, as myelin's role in neuronal circuit development expands, so too does the need for powerful models to test mechanistic hypotheses. One such resource—the larval zebrafish vestibular system—balances accessibility and sensitivity in an evolutionarily conserved context. There, one can leverage the zebrafish's cutting-edge genetic and optical technologies with the careful and quantitative measurements and models of behavior so familiar to vestibular neuroscientists. We are optimistic that this synergy will allow meaningful progress toward understanding the functional impact of myelination during normal development. We conclude with the hope that such a tool will ultimately prove its worth by informing ameliorative approaches to demyelinating disease.

## Data Availability Statement

The original contributions presented in the study are included in the article/supplementary material, further inquiries can be directed to the corresponding author/s.

## Author Contributions

FA and DS: conceptualization and editing. FA: writing. DS: funding acquisition and supervision. All authors contributed to the article and approved the submitted version.

## Funding

This work was supported by the National Institute on Deafness and Communication Disorders of the National Institutes of Health under award numbers DC017489.

## Conflict of Interest

The authors declare that the research was conducted in the absence of any commercial or financial relationships that could be construed as a potential conflict of interest.

## Publisher's Note

All claims expressed in this article are solely those of the authors and do not necessarily represent those of their affiliated organizations, or those of the publisher, the editors and the reviewers. Any product that may be evaluated in this article, or claim that may be made by its manufacturer, is not guaranteed or endorsed by the publisher.
